# Discovery of novel RNA viruses through analysis of fungi-associated next-generation sequencing data

**DOI:** 10.1186/s12864-024-10432-w

**Published:** 2024-05-27

**Authors:** Xiang Lu, Ziyuan Dai, Jiaxin Xue, Wang Li, Ping Ni, Juan Xu, Chenglin Zhou, Wen Zhang

**Affiliations:** 1 Institute of Critical Care Medicine, The Affiliated People’s Hospital, Jiangsu University, Zhenjiang, 212002 China; 2 Department of Microbiology, School of Medicine, Jiangsu University, Zhenjiang, 212013 China; 3 Department of Clinical Laboratory, Affiliated Hospital 6 of Nantong University, Yancheng Third People’s Hospital, Yancheng, Jiangsu China; 4 Clinical Laboratory Center, The Affiliated Taizhou People’s Hospital of Nanjing Medical University, Taizhou, 225300 China

**Keywords:** Fungi, Mycovirus, Virus, RNA, Evolution

## Abstract

**Background:**

Like all other species, fungi are susceptible to infection by viruses. The diversity of fungal viruses has been rapidly expanding in recent years due to the availability of advanced sequencing technologies. However, compared to other virome studies, the research on fungi-associated viruses remains limited.

**Results:**

In this study, we downloaded and analyzed over 200 public datasets from approximately 40 different Bioprojects to explore potential fungal-associated viral dark matter. A total of 12 novel viral sequences were identified, all of which are RNA viruses, with lengths ranging from 1,769 to 9,516 nucleotides. The amino acid sequence identity of all these viruses with any known virus is below 70%. Through phylogenetic analysis, these RNA viruses were classified into different orders or families, such as *Mitoviridae*, *Benyviridae*, *Botourmiaviridae*, *Deltaflexiviridae*, *Mymonaviridae*, *Bunyavirales*, and *Partitiviridae*. It is possible that these sequences represent new taxa at the level of family, genus, or species. Furthermore, a co-evolution analysis indicated that the evolutionary history of these viruses within their groups is largely driven by cross-species transmission events.

**Conclusions:**

These findings are of significant importance for understanding the diversity, evolution, and relationships between genome structure and function of fungal viruses. However, further investigation is needed to study their interactions.

**Supplementary Information:**

The online version contains supplementary material available at 10.1186/s12864-024-10432-w.

## Introduction

Viruses are among the most abundant and diverse biological entities on Earth; they are ubiquitous in the natural environment but difficult to culture and detect [1–3]. In recent decades, the significant advancements in omics have transformed the field of virology and enabled researchers to detect potential viruses in a variety of environmental samples, helping us to expand the known diversity of viruses and explore the “dark matter” of viruses that may exist in vast quantities [4]. In most cases, the hosts of these newly discovered viruses exhibit only asymptomatic infections [5, 6], and they even play an important role in maintaining the balance, stability, and sustainable development of the biosphere [7]. But some viruses may be involved in the emergence and development of animal or plant diseases. For example, the tobacco mosaic virus (TMV) causes poor growth in tobacco plants, while norovirus is known to cause diarrhea in mammals [8, 9]. In the field of fungal research, viral infections have significantly reduced the yield of edible fungi, thereby attracting increasing attention to fungal diseases caused by viruses [10]. However, due to their apparent relevance to health [11], fungal-associated viruses have been understudied compared to viruses affecting humans, animals, or plants.

Mycoviruses (also known as fungal viruses) are widely distributed in various fungi and fungal-like organisms [12]. The first mycoviruses were discovered in the 1960s by Hollings M in the basidiomycete *Agaricus bisporus*, an edible cultivated mushroom [13]. Shortly thereafter, Ellis LF et al. reported mycoviruses in the ascomycete *Penicillium stoloniferum*, confirming that viral dsRNA is responsible for interferon stimulation in mammals [13–15]. In recent years, the diversity of known mycoviruses has rapidly increased with the development and widespread application of sequencing technologies [16–20]. According to the classification principles of the International Committee for the Taxonomy of Viruses (ICTV), mycoviruses are currently classified into 24 taxa, consisting of 23 families and 1 genus (*Botybirnavirus*) [21]. Most mycoviruses belong to double-stranded (ds) RNA viruses, such as families *Totiviridae*, *Partitiviridae*, *Reoviridae*, *Chrysoviridae*, *Megabirnaviridae*, *Quadriviridae*, and genus *Botybirnavirus*, or positive-sense single-stranded (+ ss) RNA viruses, such as families *Alphaflexiviridae*, *Gammaflexiviridae*, *Barnaviridae*, *Hypoviridae*, *Endornaviridae*, *Metaviridae* and *Pseudoviridae*. However, negative-sense single-stranded (-ss) RNA viruses (family *Mymonaviridae*) and single-stranded (ss) DNA viruses (family *Genomoviridae*) have also been described [22]. The taxonomy of mycoviruses is continually refined as novel mycoviruses that cannot be classified into any established taxon are identified. While the vast majority of fungi-infecting viruses do not show infection characteristics and have no significant impact on their hosts, some mycoviruses have inhibitory effects on the phenotype of the host, leading to hypovirulence in phytopathogenic fungi [23]. The use of environmentally friendly, low-virulence-related mycoviruses such as Chryphonectria hypovirus 1 (CHV-1) for biological control has been considered a viable alternative to chemical fungicides [24]. With the deepening of research, an increasing number of mycoviruses that can cause fungal phenotypic changes have been identified [3, 23, 25]. Therefore, understanding the distribution of these viruses and their effects on hosts will allow us to determine whether their infections can be prevented and treated.

To explore the viral dark matter hidden within fungi, this study collected over 200 available fungal-associated libraries from approximately 40 Bioprojects in the Sequence Read Archive (SRA) database, uncovering novel RNA viruses within them. We further elucidated the genetic relationships between known viruses and these newfound ones, thereby expanding our understanding of fungal-associated viruses and providing assistance to viral taxonomy.

## Materials and methods

### Genome assembly

To discover novel fungal-associated viruses, we downloaded 236 available libraries from the SRA database, corresponding to 32 fungal species (Supplementary Table 1). Pfastq-dump v0.1.6 (https://github.com/inutano/pfastq-dump) was used to convert SRA format files to fastq format files. Subsequently, Bowtie2 v2.4.5 [26] was employed to remove host sequences. Primer sequences of raw reads underwent trimming using Trim Galore v0.6.5 (https://www.bioinformatics.babraham.ac.uk/projects/trim_galore), and the resulting files underwent quality control with the options ‘–phred33 –length 20 –stringency 3 –fastqc’. Duplicated reads were marked using PRINSEQ-lite v0.20.4 (-derep 1). All SRA datasets were then assembled in-house pipeline. Paired-end reads were assembled using SPAdes v3.15.5 [27] with the option ‘-meta’, while single-end reads were assembled with MEGAHIT v1.2.9 [28], both using default parameters. The results were then imported into Geneious Prime v2022.0.1 (https://www.geneious.com) for sorting and manual confirmation. To reduce false negatives during sequence assembly, further semi-automatic assembly of unmapped contigs and singlets with a sequence length < 500 nt was performed. Contigs with a sequence length > 1,500 nt after reassembly were retained. Individual contigs were then used as references for mapping to the raw data using the Low Sensitivity/Fastest parameter in Geneious Prime. In addition, mixed assembly was performed using MEGAHIT in combination with BWA v0.7.17 [29] to search for unused reads that might correspond to low-abundance contigs.

### Searching for novel viruses in fungal libraries

We identified novel viral sequences present in fungal libraries through a series of steps. To start, we established a local viral database, consisting of the non-redundant protein (nr) database downloaded in August 2023, along with IMG/VR v3 [30], for screening assembled contigs. The contigs labeled as “viruses” and exhibiting less than 70% amino acid (aa) sequence identity with the best match in the database were imported into Geneious Prime for manual mapping. Putative open reading frames (ORFs) were predicted by Geneious Prime using built-in parameters (Minimum size: 100) and were subsequently verified by comparison to related viruses. The annotations of these ORFs were based on comparisons to the Conserved Domain Database (CDD). The sequences after manual examination were subjected to genome clustering using MMseqs2 (-k 0 -e 0.001 –min-seq-id 0.95 -c 0.9 –cluster-mode 0) [31]. After excluding viruses with high aa sequence identity (> 70%) to known viruses, a dataset containing a total of 12 RNA viral sequences was obtained. The non-redundant fungal virus dataset was compared against the local database using the BLASTx program built in DIAMOND v2.0.15 [32], and significant sequences with a cut-off E-value of < 10^–5^ were selected. The coverage of each sequence in all libraries was calculated using the pileup tool in BBMap. Taxonomic identification was conducted using TaxonKit [33] software, along with the rma2info program integrated into MEGAN6 [34]. The RNA secondary structure prediction of the novel viruses was conducted using RNA Folding Form V2.3 (http://www.unafold.org/mfold/applications/rna-folding-form-v2.php).

### Phylogenetic analysis

To infer phylogenetic relationships, nucleotide and their encoded protein sequences of reference strains belonging to different groups of corresponding viruses were downloaded from the NCBI GenBank database, along with sequences of proposed species pending ratification. Related sequences were aligned using the alignment program within the CLC Genomics Workbench 10.0, and the resulting alignment was further optimized using MUSCLE in MEGA-X [35]. Sites containing more than 50% gaps were temporarily removed from the alignments. Maximum-likelihood (ML) trees were then constructed using IQ-TREE v1.6.12 [36]. All phylogenetic trees were created using IQ-TREE with 1,000 bootstrap replicates (-bb 1000) and the ModelFinder function (-m MFP). Interactive Tree Of Life (iTOL) was used for visualizing and editing phylogenetic trees [37]. Colorcoded distance matrix analysis between novel viruses and other known viruses were performed with Sequence Demarcation Tool v1.2 [38].

To illustrate cross-species transmission and co-divergence between viruses and their hosts across different virus groups, we reconciled the co-phylogenetic relationships between these viruses and their hosts. The evolutionary tree and topologies of the hosts involved in this study were obtained from the TimeTree [39] website by inputting their Latin names. The viruses in the phylogenetic tree for which the host cannot be recognized through published literature or information provided by the authors are disregarded. The co-phylogenetic plots (or ‘tanglegram’) generated using the R package phytools [40] visually represent the correspondence between host and virus trees, with lines connecting hosts and their respective viruses. The event-based program eMPRess [41] was employed to determine whether the pairs of virus groups and their hosts undergo coevolution. This tool reconciles pairs of phylogenetic trees according to the Duplication-Transfer-Loss (DTL) model [42], employing a maximum parsimony formulation to calculate the cost of each coevolution event. The cost of duplication, host-jumping (transfer), and extinction (loss) event types were set to 1.0, while host-virus co-divergence was set to zero, as it was considered the null event.

### Data availability

The data reported in this paper have been deposited in the GenBase in National Genomics Data Center [43], Beijing Institute of Genomics, Chinese Academy of Sciences/China National Center for Bioinformation, under accession numbers C_AA066339.1-C_AA066350.1 that are publicly accessible at https://ngdc.cncb.ac.cn/genbase. Please refer to Table 1 for details.

**Table 1:** Assembled sequences with identity to those of previously described viruses

Virus name	Accession	Length	Best Match	aa Identity	Virus Family	E-value	Query cover	Mapping reads
TeMV01	C_AA066349.1	2,689	Ophiostoma mitovirus 4 [NP_660179]	51.47%	*Mitoviridae*	0	82%	35,248
TeMV02	C_AA066342.1	3,087	Plasmopara viticola lesion associated mitovirus 40 [QIR30263]	42.82%	*Mitoviridae*	2e-142	69%	58,505
GtBeV	C_AA066339.1	6,479	Diabrotica undecimpunctata virus 2 [QIT20101]	34.68%	*Benyviridae*	2e-123	77%	58,149
CrBV	C_AA066344.1	2,903	Erysiphe necator associated ourmia-like virus 2 [UUW21020]	56.58%	*Botourmiaviridae*	0	69%	309,690
LsDV	C_AA066341.1	3,425	Cat Tien Macrotermes Deltaflexi-like virus [UUW06602]	46.61%	*Deltaflexiviridae*	0	94%	47,164
GtTIV	C_AA066348.1	7,588	Fusarium sacchari alphavirus-like virus 1 [QIQ28421]	60.43%	Unclassified	0	97%	1,124
GtMV	C_AA066347.1	9,339	Soybean leaf-associated negative-stranded RNA virus 2 [YP_010784557]	45.22%	*Mymonaviridae*	0	95%	43,440
ApMV	C_AA066346.1	6,235	Erysiphe necator associated negative-stranded RNA virus 23 [YP_010802816]	55.90%	*Mymonaviridae*	0	94%	29,691
CoBV	C_AA066345.1	7,277	Suillus luteus associated bunya-like virus 2 [WLK77441]	32.97%	*Phasmaviridae*	0	80%	1,465
GtBV	C_AA066343.1	7,364	Entoleuca phenui-like virus 1 [YP_010086241]	54.20%	*Phenuiviridae*	0	95%	839
TaBV	C_AA066340.1	9,516	Trichoderma gamsii mycobunyavirus 1 [WGH72967]	35.02%	Unclassified	0	75%	2,914
NcPV	C_AA066350.1	1,769	Pythium nunn virus 1 [YP_009551507]	41.50%	*Partitiviridae*	2e-120	84%	13,506

## Results

### Twelve novel RNA viruses associated with *fungi*

We investigated fungi-associated novel viruses by mining publicly available metagenomic and transcriptomic fungal datasets. In total, we collected 236 datasets, which were categorized into four fungal phyla: *Ascomycota* (159), *Basidiomycota* (47), *Chytridiomycota* (15), and *Zoopagomycota* (15). These phyla corresponded to 20, 8, 2, and 2 different fungal genera, respectively (Supplementary Table 1). A total of 12 sequences containing complete coding DNA sequences (CDS) for RNA-dependent RNA polymerase (RdRp) have been identified, ranging in length from 1,769 nt to 9,516 nt. All of these sequences have less than 70% aa identity with RdRp sequences from any currently known virus (ranging from 32.97% to 60.43%), potentially representing novel families, genera, or species (Table 1). Some of the identified sequences were shorter than the reference genomes of RNA viruses, suggesting that these viral sequences represented partial sequences of viral genomes. To exclude the possibility of transient viral infections in hosts or de novo assembly artefacts in co-infection detection, we extracted the nucleotide sequences of the coding regions of these 12 sequences and mapped them to all collected libraries to compute coverage (Supplementary Table 2). The results revealed varying degrees of read matches for these viral genomes across different libraries, spanning different fungal species. Although we only analyzed sequences longer than 1,500 nt, it is worth noting that we also discovered other viral reads in many libraries. However, we were unable to assemble them into sufficiently long contigs, possibly due to library construction strategies or sequencing depth. In any case, this preliminary finding reveals a greater diversity of fungal-associated viruses than previously considered.

### Positive-sense single-stranded RNA viruses

#### (i) Mitoviridae

**Fig. 1:**
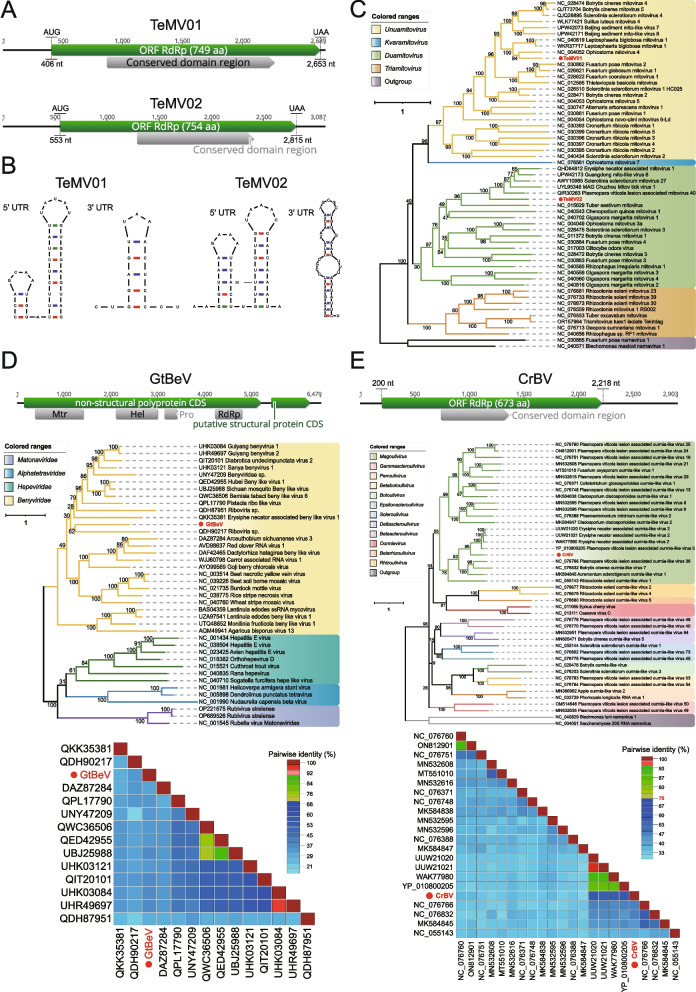
Identification of novel positive-sense single-stranded RNA viruses in fungal sequencing libraries. **A** Genome organization of two novel mitoviruses; the putative ORF for the viral RdRp is depicted by a green box, and the predicted conserved domain region is displayed in a gray box. **B** Predicted RNA secondary structures of the 5'- and 3'-terminal regions. **C** ML phylogenetic tree of members of the family *Mitoviridae*. The best-fit model (LG + F + R6) was estimated using IQ-Tree model selection. The bootstrap value is shown at each branch, with the newly identified viruses represented in red font. **D** The genome organization of GtBeV is depicted at the top; in the middle is the ML phylogenetic tree of members of the family *Benyviridae*. The best-fit model (VT + F + R5) was estimated using IQ-Tree model selection. The bootstrap value is shown at each branch, with the newly identified virus represented in red font. At the bottom is the distance matrix analysis of GeBeV identified in *Gaeumannomyces tritici*. Pairwise sequence comparison produced with the RdRp amino acid sequences within the ML tree. **E** The genome organization of CrBV is depicted at the top; in the middle is the ML phylogenetic tree of members of the family *Botourmiaviridae*. The best-fit model (VT + F + R5) was estimated using IQ-Tree model selection. The bootstrap value is shown at each branch, with the newly identified virus represented in red font. At the bottom is the distance matrix analysis of CrBV identified in *Clonostachys rosea*. Pairwise sequence comparison produced with the RdRp amino acid sequences within the ML tree

Members of the family *Mitoviridae* (order *Cryppavirales*) are monopartite, linear, positive-sense ( +) single-stranded (ss) RNA viruses with genome size of approximately 2.5–2.9 kb [44], carrying a single long open reading frame (ORF) which encodes a putative RdRp. Mitoviruses have no true virions and no structural proteins, virus genome is transmitted horizontally through mating or vertically from mother to daughter cells [45]. They use mitochondria as their sites of replication and have typical 5' and 3' untranslated regions (UTRs) of varying sizes, which are responsible for viral translation and replicase recognition [46]. According to the taxonomic principles of ICTV, the viruses belonging to the family *Mitoviridae* are divided into four genera, namely *Duamitovirus*, *Kvaramitovirus*, *Triamitovirus* and *Unuamitovirus*. In this study, two novel viruses belonging to the family *Mitoviridae* were identified in the same library (SRR12744489; Species: *Thielaviopsis ethacetica*), named *Thielaviopsis ethacetica* mitovirus 1 (TeMV01) and *Thielaviopsis ethacetica* mitovirus 2 (TeMV02), respectively (Fig. 1A). The genome sequence of TeMV01 spans 2,689 nucleotides in length with a GC content of 32.2%. Its 5' and 3' UTRs comprise 406 nt and 36 nt, respectively. Similarly, the genome sequence of TeMV02 extends 3,087 nucleotides in length with a GC content of 32.6%. Its 5' and 3' UTRs consist of 553 and 272 nt, respectively. The 5' and 3' ends of both genomes are predicted to have typical stem-loop structures (Fig. 1B). In order to determine the evolutionary relationship between these two mitoviruses and other known mitoviruses, phylogenetic analysis based on RdRp showed that viral strains were divided into 2 genetic lineages in the genera *Duamitovirus* and *Unuamitovirus* (Fig. 1C). In the genus *Unuamitovirus*, TeMV01 was clustered with Ophiostoma mitovirus 4, exhibiting the highest aa identity of 51.47%, while in the genus *Duamitovirus*, TeMV02 was clustered with a strain isolated from *Plasmopara viticola*, showing the highest aa identity of 42.82%. According to the guidelines from the ICTV regarding the taxonomy of the family *Mitoviridae*, a species demarcation cutoff of < 70% aa sequence identity is established [47]. Drawing on this recommendation and phylogenetic inferences, these two viral strains could be presumed to be novel viral species [48].

#### (ii) Benyviridae

The family *Benyviridae* is comprised of multipartite plant viruses that are rod-shaped, approximately 85–390 nm in length and 20 nm in diameter. Within this family, there is a single genus, *Benyvirus* [49]. It is reported that one species within this genus,Beet necrotic yellow vein virus, can cause widespread and highly destructive soil-borne ‘rhizomania’ disease of sugar beet [50]. A full-length RNA1 sequence related to *Benyviridae* has been detected from *Gaeumannomyces tritici* (ERR3486062), with a length of 6,479 nt. It possesses a poly(A) tail at the 3' end and is temporarily designated as *Gaeumannomyces tritici* benyvirus (GtBeV). BLASTx results indicate a 34.68% aa sequence identity with the best match found (Fig. 1D). The non-structural polyprotein CDS of RNA1 encodes a large replication-associated protein of 1,688 amino acids with a molecular mass of 190 kDa. Four domains were predicted in this polyprotein corresponding to representative species within the family *Benyviridae*. The viral methyltransferase (Mtr) domain spans from nucleotide position 386 to 1411, while the RNA helicase (Hel) domain occupies positions 2113 to 2995 nt. Additionally, the protease (Pro) domain is located between positions 3142 and 3410 nt, and the RdRp domain is located at 4227 to 4796 nt. A phylogenetic analysis was conducted by integrating RdRp sequences of viruses closely related to GtBeV. The result revealed that GtBeV clustered within the family *Benyviridae*, exhibiting substantial evolutionary divergence from any other sequences. Consequently, this virus likely represents a novel species in the family *Benyviridae*.

#### (iii) Botourmiaviridae

The family *Botourmiaviridae* comprises viruses infecting plants and filamentous fungi, which may possess mono- or multi-segmented genomes [51]. Recent research has led to a rapid expansion in the number of viruses within the family *Botourmiaviridae*, increasing from the confirmed 4 genera in 2020 to a total of 12 genera. A contig identified from *Clonostachys rosea* (ERR5928658) using the BLASTx method exhibited similarity to viruses in the family *Botourmiaviridae*. After manual mapping, a 2,903 nt-long genome was obtained, tentatively named *Clonostachys rosea* botourmiavirus (CrBV), which includes a complete RdRP region (Fig. 1E). Based on phylogenetic analysis using RdRp, CrBV clustered with members of the genus *Magoulivirus*, sharing 56.58% aa identity with a strain identified from *Eclipta prostrata*. However, puzzlingly, according to the ICTV's Genus/Species demarcation criteria, members of different genera/species within the family *Botourmiaviridae* share less than 70%/90% identity in their complete RdRP amino acid sequences. Furthermore, the RdRp sequences with accession numbers NC_055143 and NC_076766, both considered to be members of the genus *Magoulivirus*, exhibited only 39.05% aa identity to each other. Therefore, CrBV should at least be considered as a new species within the family *Botourmiaviridae*.

#### (iv) Deltaflexiviridae

**Fig. 2:**
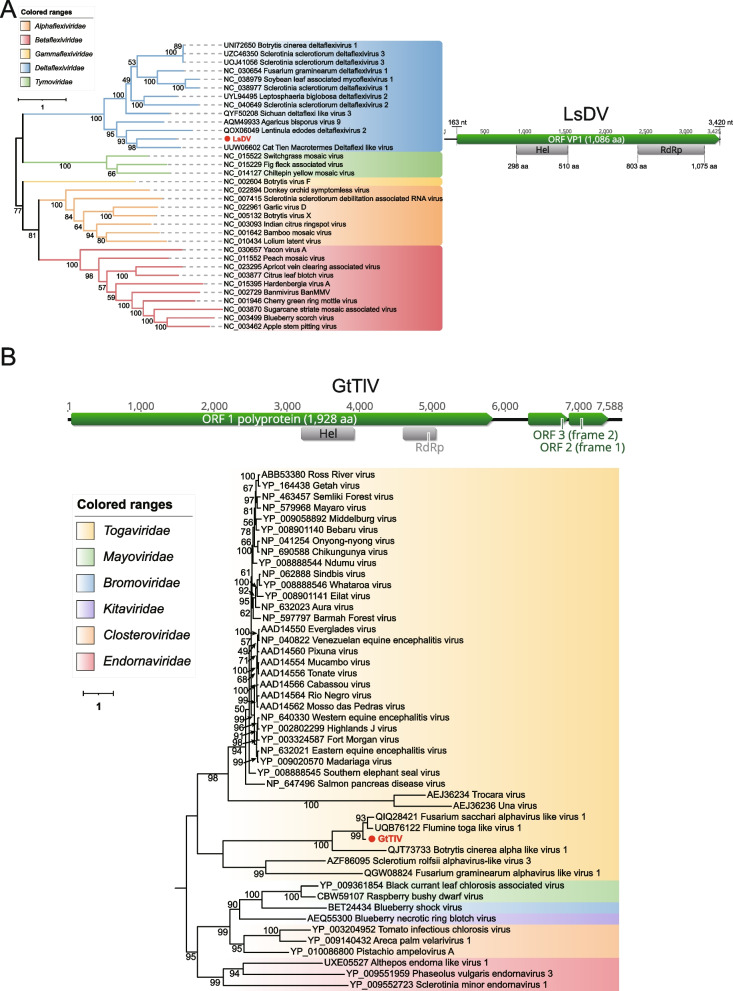
Identification of novel members of family *Deltaflexiviridae* and Toga-like virus in fungal sequencing libraries. **A** On the right side of the image is the genome organization of LsDV; the putative ORF for the viral RdRp is depicted by a green box, and the predicted conserved domain region is displayed in a gray box. ML phylogenetic tree of members of the family *Deltaflexiviridae*. The best-fit model (VT + F + R6) was estimated using IQ-Tree model selection. The bootstrap value is shown at each branch, with the newly identified virus represented in red font. **B** The genome organization of GtTlV is depicted at the top; the putative ORF for the viral RdRp is depicted by a green box, and the predicted conserved domain region is displayed in a gray box. ML phylogenetic tree of members of the order *Martellivirales*. The best-fit model (LG + R7) was estimated using IQ-Tree model selection. The bootstrap value is shown at each branch, with the newly identified virus represented in red font

An assembled sequence of 3,425 nucleotides in length *Lepista sordida* deltaflexivirus (LsDV), derived from *Lepista sordida* (DRR252167) and showing homology to *Deltaflexiviridae* within the order *Tymovirales*, was obtained. The *Tymovirales* comprises five recognized families: *Alphaflexiviridae*, *Betaflexiviridae*, *Deltaflexiviridae*, *Gammaflexiviridae*, and *Tymoviridae* [52]. The *Deltaflexiviridae* currently only includes one genus, the fungal-associated deltaflexivirus; they are mostly identified in fungi or plants pathogens [53]. LsDV was predicted to have a single large ORF, VP1, which starts with an AUG codon at nt 163–165 and ends with a UAG codon at nt 3,418–3,420. This ORF encodes a putative polyprotein of 1,086 aa with a calculated molecular mass of 119 kDa. Two conserved domains within the VP1 protein were identified: Hel and RdRp (Fig. 2A). However, the Mtr was missing, indicating that the 5' end of this polyprotein is incomplete. According to the phylogenetic analysis of RdRp, LsDV was closely related to viruses of the family *Deltaflexiviridae* and shared 46.61% aa identity with a strain (UUW06602) isolated from *Macrotermes carbonarius*. Despite this, according to the species demarcation criteria proposed by ICTV, because we couldn't recover the entire replication-associated polyprotein, LsDV cannot be regarded as a novel species at present.

#### (v) Toga-like virus

Members of the family *Togaviridae* are primarily transmitted by arthropods and can infect a wide range of vertebrates, including mammals, birds, reptiles, amphibians, and fish [54]. Currently, this family only contains a single confirmed genus, *Alphavirus*. A contig was discovered in *Gaeumannomyces tritici* (ERR3486058), it is 7,588 nt in length with a complete ORF encoding a putative protein of 1,928 aa, which had 60.43% identity to Fusarium sacchari alphavirus-like virus 1 (QIQ28421) with 97% coverage. Phylogenetic analysis showed that it did not cluster with classical alphavirus members such as VEE, WEE, EEE, SF complex [54], but rather with several sequences annotated as Toga-like that were available (Fig. 2B). It was provisionally named *Gaeumannomyces tritici* toga-like virus (GtTIV). However, we remain cautious about the accuracy of these so-called Toga-like sequences, as they show little significant correlation with members of the order *Martellivirales*.

### Negative-sense single-stranded RNA viruses

#### (i) Mymonaviridae

**Fig. 3:**
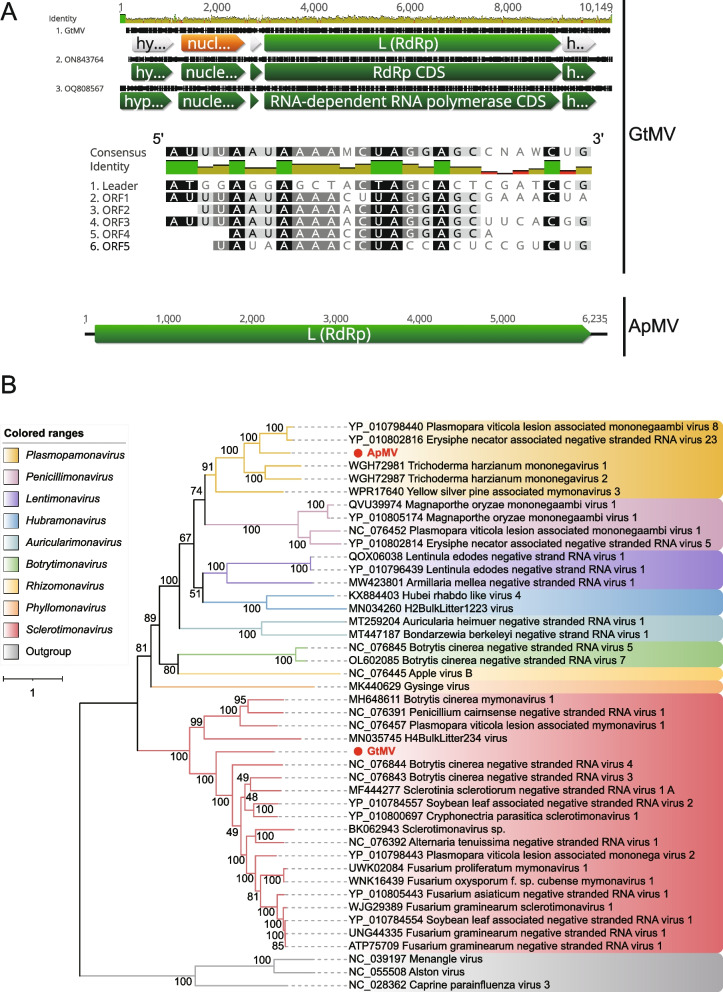
Identification of two new members in the family *Mymonaviridae*. **A** At the top is the nucleotide multiple sequence alignment result of GtMV with the reference genomes. the putative ORF for the viral RdRp is depicted by a green box, the predicted nucleoprotein is displayed in a yellow box, and three hypothetical proteins are displayed in gray boxes. The comparison of putative semi-conserved regions between ORFs in GtMV is displayed in the 5' to 3' orientation, with conserved sequences are highlighted. At the bottom is the genome organization of AmPV; the putative ORF for the viral RdRp is depicted by a green box. **B** ML phylogenetic tree of members of the family *Mymonaviridae*. The best-fit model (LG + F + R6) was estimated using IQ-Tree model selection. The bootstrap value is shown at each branch, with the newly identified viruses represented in red font

*Mymonaviridae* is a family of linear, enveloped, negative-stranded RNA genomes in the order *Mononegavirales*, which infect fungi. They are approximately 10 kb in size and encode six proteins [55]. The famliy *Mymonaviridae* was established to accommodate *Sclerotinia sclerotiorum negative-stranded RNA virus 1* (SsNSRV-1), a novel virus discovered in a hypovirulent strain of *Sclerotinia sclerotiorum* [56]. According to the ICTV, the family *Mymonaviridae* currently includes 9 genera, namely *Auricularimonavirus*, *Botrytimonavirus*, *Hubramonavirus*, *Lentimonavirus*, *Penicillimonavirus*, *Phyllomonavirus*, *Plasmopamonavirus*, *Rhizomonavirus* and *Sclerotimonavirus*. Two sequences originating from *Gaeumannomyces tritici* (ERR3486068) and *Aspergillus puulaauensis* (DRR266546), respectively, and associated with the family *Mymonaviridae*, have been identified and provisionally named *Gaeumannomyces tritici* mymonavirus (GtMV) and *Aspergillus puulaauensis* mymonavirus (ApMV). GtMV is 9,339 nt long with a GC content of 52.8%. It was predicted to contain 5 discontinuous ORFs, with the largest one encoding RdRp. Additionally, a nucleoprotein and three hypothetical proteins with unknown function were also predicted. A multiple alignment of nucleotide sequences among these ORFs identified a semi-conserved sequence, 5'-UAAAA-CUAGGAGC-3', located downstream of each ORF (Fig. 3A). These regions are likely gene-junction regions in the GtMV genome, a characteristic feature shared by mononegaviruses [57, 58]. For ApMV, a complete RdRp CDS with a length of 1,978 aa was predicted. The BLASTx searches showed that GtMV shared 45.22% identity with the RdRp of Soybean leaf-associated negative-stranded RNA virus 2 (YP_010784557), while ApMV shared 55.90% identity with the RdRp of Erysiphe necator associated negative-stranded RNA virus 23 (YP_010802816). The representative members of the family *Mymonaviridae* were included in the phylogenetic analysis. The results showed that GtMV and ApMV clustered closely with members of the genera *Sclerotimonavirus* and *Plasmopamonavirus*, respectively (Fig. 3B). Members of the genus *Plasmopamonavirus* are about 6 kb in size and encode for a single protein. Therefore, GtMV and ApMV should be considered as representing new species within their respective genera.

#### (ii) *Bunyavirales*

**Fig. 4:**
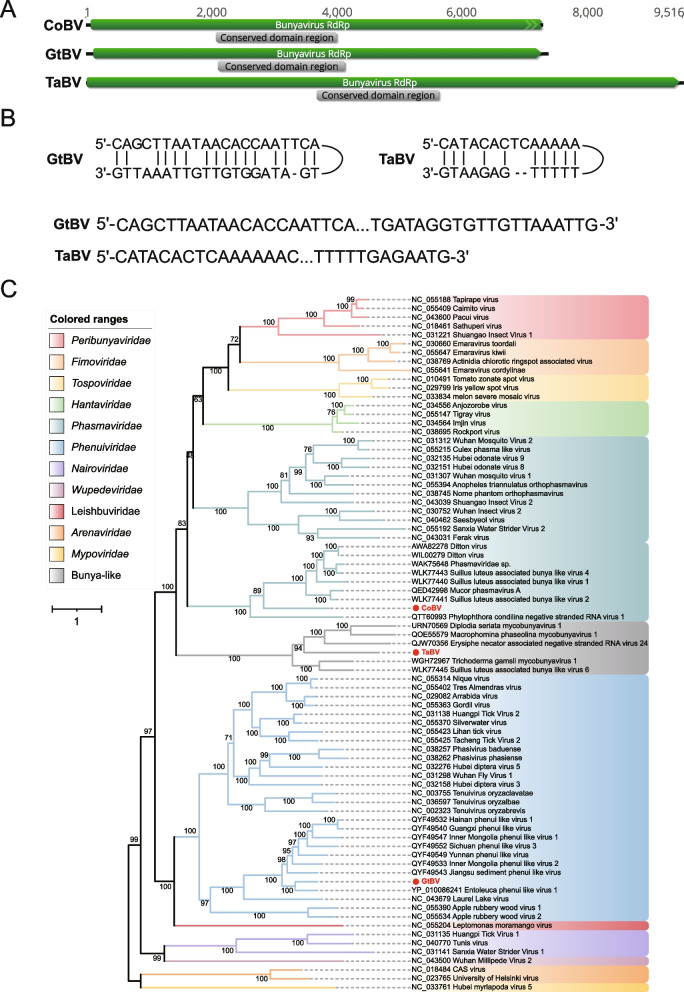
Identification of three new members in the order *Bunyavirales*. **A** The genome organization of CoBV, GtBV, and TaBV; the putative ORF for the viral RdRp is depicted by a green box, and the predicted conserved domain region is displayed in a gray box. **B** The complementary structures formed at the 5' and 3' ends of GtBV and TaBV. **C** ML phylogenetic tree of members of the order *Bunyavirales*. The best-fit model (VT + F + R8) was estimated using IQ-Tree model selection. The bootstrap value is shown at each branch, with the newly identified viruses represented in red font

The *Bunyavirales* (the only order in the class *Ellioviricetes*) is one of the largest groups of segmented negative-sense single-stranded RNA viruses with mainly tripartite genomes [59], which includes many pathogenic strains that infect arthropods(such as mosquitoes, ticks, sand flies), plants, protozoans, and vertebrates, and even cause severe human diseases. Order *Bunyavirales* consists of 14 viral families, including *Arenaviridae*, *Cruliviridae*, *Discoviridae*, *Fimoviridae*, *Hantaviridae*, *Leishbuviridae*, *Mypoviridae*, *Nairoviridae*, *Peribunyaviridae*, *Phasmaviridae*, *Phenuiviridae*, *Tospoviridae*, *Tulasviridae* and *Wupedeviridae*. In this study, three complete or near complete RNA1 sequences related to bunyaviruses were identified and named according to their respective hosts: CoBV (*Conidiobolus obscurus* bunyavirus; SRR6181013; 7,277 nt), GtBV (*Gaeumannomyces tritici* bunyavirus; ERR3486069; 7,364 nt), and TaBV (*Thielaviopsis aethacetica* bunyavirus; SRR12744489; 9,516 nt) (Fig. 4A). The 5' and 3' terminal RNA segments of GtBV and TaBV complement each other, allowing the formation of a panhandle structure [60], which plays an essential role as promoters of genome transcription and replication [61], except for CoBV, as the 3' terminal of CoBV has not been fully obtained (Fig. 4B). BLASTx results indicated that these three viruses had identities ranging from 32.97% to 54.20% to the best matches in the GenBank database. Phylogenetic analysis indicated that CoBV was classified into the family *Phasmaviridae*, with distant relationships to any of its genera; GtBV clustered well with members of the genus *Entovirus* of family *Phenuiviridae*; while TaBV did not cluster with any known members of families within *Bunyavirales*, hence provisionally placed within the Bunya-like group (Fig. 4C). Therefore, these three sequences should be considered as potential new family, genus, or species within the order *Bunyavirales*.

### Double-stranded RNA viruses

#### Partitiviridae

**Fig. 5:**
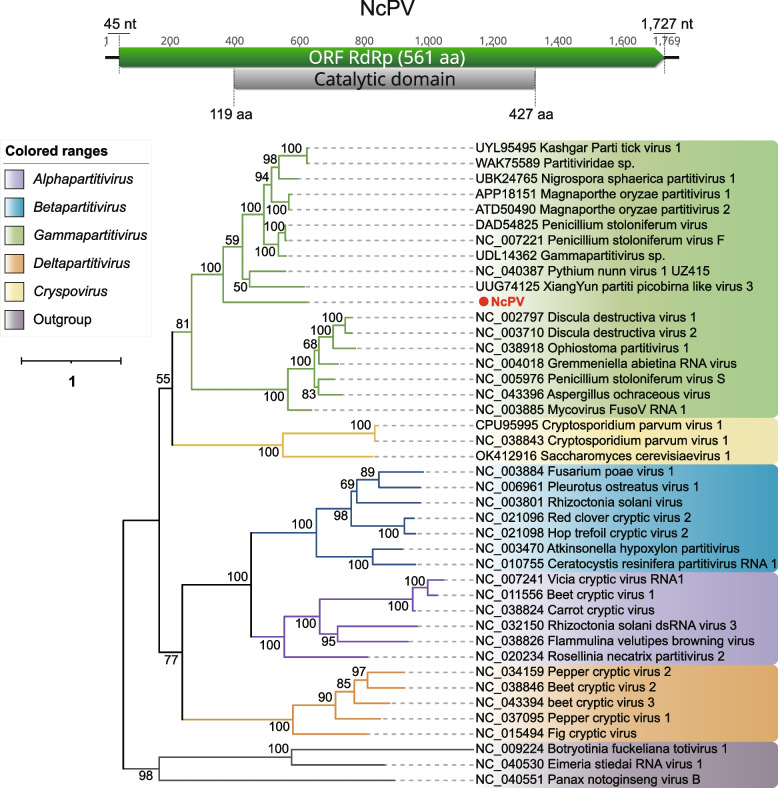
Identification of a new member in the family *Partitiviridae*. The genome organization of NcPV is depicted at the top; the putative ORF for the viral RdRp is depicted by a green box, and the predicted conserved domain region is displayed in a gray box. At the bottom is the ML phylogenetic tree of members of the family *Partitiviridae*. The best-fit model (VT + F + R4) was estimated using IQ-Tree model selection. The bootstrap value is shown at each branch, with the newly identified virus represented in red font

The *Partitiviridae* is a family of small, non-enveloped viruses, approximately 35–40 nm in diameter, with bisegmented double-stranded (ds) RNA genomes. Each segment is about 1.4–3.0 kb in size, resulting in a total size about 4 kb [62]. The family *Partitiviridae* is now divided into five genera: *Alphapartitivirus*, *Betapartiivirus*, *Cryspovirus*, *Deltapartitivirus* and *Gammapartitivirus*. Each genus has characteristic hosts: plants or fungi for *Alphapartitivirus* and *Betapartitivirus*, fungi for *Gammapartitivirus*, plants for *Deltapartitivirus*, and protozoa for *Cryspovirus* [62]. A complete dsRNA1 sequence *Neocallimastix californiae* partitivirus (NcPV) retrieved from *Neocallimastix californiae* (SRR15362281) has been identified as being associated with the family *Partitiviridae*. The BLASTp result indicated that it shared the highest aa identity of 41.5% with members of the genus *Gammapartitivirus*. According to the phylogenetic tree constructed based on RdRp, NcPV was confirmed to fall within the genus *Gammapartitivirus* (Fig. 5). Typical members of the genus *Gammapartitivirus* have two segments in their complete genome, namely dsRNA1 and dsRNA2, encoding RdRp and coat protein, respectively [62]. The larger dsRNA1 segment of NcPV measures 1,769 nt in length, with a GC content of 35.8%. It contains a single ORF encoding a 561 aa RdRp. A CDD search revealed that the RdRp of NcPV harbors a catalytic region spanning from 119 to 427aa. Regrettably, only the complete dsRNA1 segment was obtained. According to the classification principles of ICTV, due to the lack of information regarding dsRNA2, we are unable to propose it as a new species. It is worth noting that according to the Genus demarcation criteria (https://ictv.global/report/chapter/partitiviridae/partitiviridae), members of the genus *Gammapartitivirus* should have a dsRNA1 length ranging from 1645 to 1787 nt, and the RdRp length should fall between 519 and 539 aa. However, the length of dsRNA1 in NcPV is 1,769 nt, with RdRp being 561 aa, challenging this classification criterion. In fact, multiple strains have already exceeded this criterion, such as GenBank accession numbers: WBW48344, UDL14336, QKK35392, among others.

### Long-term evolutionary relationships between fungal-associated viruses and hosts

**Fig. 6:**
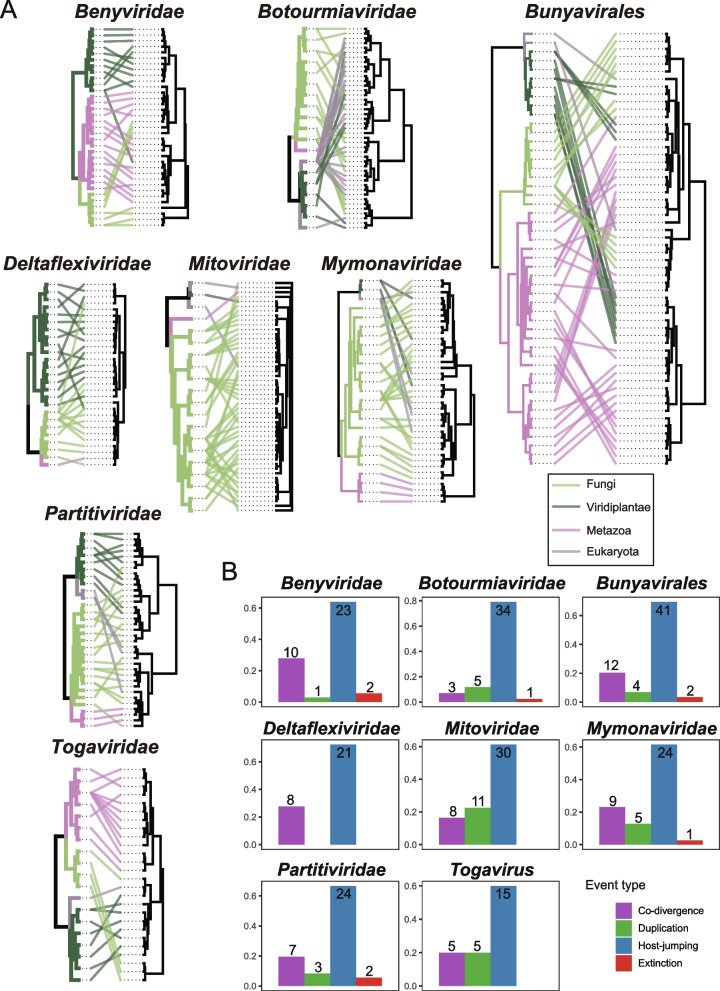
Co-evolutionary analysis of virus and host. **A** Tanglegram of phylogenetic trees for virus orders/families and their hosts. Lines and branches are color-coded to indicate host clades. The cophylo function in phytools was employed to enhance congruence between the host (left) and virus (right) phylogenies. **B** Reconciliation analysis of virus groups. The bar chart illustrates the proportional range of possible evolutionary events, with the frequency of each event displayed at the top of its respective column

Understanding the co-divergence history between viruses and hosts helps reveal patterns of virus transmission and infection and influences the biodiversity and stability of ecosystems. To explore the frequency of cross-species transmission and co-divergence among fungi-associated viruses, we constructed tanglegrams illustrating the interconnected evolutionary histories of viral families and their respective hosts through phylogenetic trees (Fig. 6A). The results indicated that cross-species transmission (Host-jumping) consistently emerged as the most frequent evolutionary event among all groups of RNA viruses examined in this study (median, 66.79%; range, 60.00% to 79.07%) (Fig. 6B). This finding is highly consistent with the evolutionary patterns of RNA viruses recently identified by Mifsud et al. in their extensive transcriptome survey of plants [63]. Members of the families *Botourmiaviridae* (79.07%) and *Deltaflexiviridae* (72.41%) were most frequently involved in cross-species transmission. The frequencies of co-divergence (median, 20.19%; range, 6.98% to 27.78%), duplication (median, 10.60%; range, 0% to 22.45%), and extinction (median, 2.42%; range, 0% to 5.56%) events involved in the evolution of fungi-associated viruses gradually decrease. Specifically, members of the family *Benyviridae* exhibited the highest frequency of co-divergence events, which also supports the findings reported by Mifsud et al.; certain studies propose that members of *Benyviridae* are transmitted via zoospores of plasmodiophorid protist [64]. It's speculated that the ancestor of these viruses underwent interkingdom horizontal transfer between plants and protists over evolutionary timelines [65]. Members of the family *Mitoviridae* showed the highest frequency of duplication events; and members of the families *Benyviridae* and *Partitiviridae* demonstrated the highest frequency of extinction events. Not surprisingly, this result is influenced by the current limited understanding of virus-host relationships. On one hand, viruses whose hosts cannot be recognized through published literature or information provided by authors have been overlooked. On the other hand, the number of viruses recorded in reference databases represents just the tip of the iceberg within the entire virosphere. The involvement of a more extensive sample size in the future should change this evolutionary landscape.

## Discussion

Our understanding of the interactions between fungi and their associated viruses has long been constrained by insufficient sampling of fungal species. Advances in metagenomics in recent decades have led to a rapid expansion of the known viral sequence space, but it is far from saturated. The diversity of hosts, the instability of the viral structures (especially RNA viruses), and the propensity to exchange genetic material with other host viruses all contribute to the unparalleled diversity of viral genomes [66]. Fungi are diverse and widely distributed in nature and are closely related to humans. A few fungi can parasitize immunocompromised humans, but their adverse effects are limited. As decomposers in the biological chain, fungi can decompose the remains of plants and animals and maintain the material cycle in the biological world [67]. In agricultural production, many fungi are plant pathogens, and about 80% of plant diseases are caused by fungi. However, little is currently known about the diversity of mycoviruses and how these viruses affect fungal phenotypes, fungal-host interactions, and virus evolution, and the sequencing depth of fungal libraries in most public databases only meets the needs of studying bacterial genomes. Sampling viruses from a larger diversity of fungal hosts should lead to new and improved evolutionary scenarios.

RNA viruses are widespread in deep-sea sediments [68], freshwater [69], sewage [70], and rhizosphere soils [71]. Compared to DNA viruses, RNA viruses are less conserved, prone to mutation, and can transfer between different hosts, potentially forming highly differentiated and unrecognized novel viruses. This characteristic increases the difficulty of monitoring these viruses. Previously, all discovered mycoviruses were RNA viruses. Until 2010, Yu et al. reported the discovery of a DNA virus, namely SsHADV-1, in fungi for the first time [72]. Subsequently, new fungal-related DNA viruses are continually being identified [73–75]. Currently, viruses have been found in all major groups of fungi, and approximately 100 types of fungi can be infected by viruses, instances exist where one virus can infect multiple fungi, or one fungus can be infected by several viruses simultaneously. The transmission of mycoviruses differs from that of animal and plant viruses and is mainly categorized into vertical and horizontal transmission [76]. Vertical transmission refers to the spread of the mycovirus to the next generation through the sexual or asexual spores of the fungus, while horizontal transmission refers to the spread of the mycovirus from one strain to another through fusion between hyphae. In the phylum *Ascomycota*, mycoviruses generally exhibit a low ability to transmit vertically through ascospores, but they are commonly transmitted vertically to progeny strains through asexual spores [77].

In this study, we identified two novel species belonging to different genera within the family *Mitoviridae*. Interestingly, they both simultaneously infect the same fungus—*Thielaviopsis ethacetica*, the causal agent of pineapple sett rot disease in sugarcane [78]. Previously, a report identified three different mitoviruses in *Fusarium circinatum* [79]. These findings suggest that there may be a certain level of adaptability or symbiotic relationship among members of the family *Mitoviridae*. Benyviruses are typically considered to infect plants, but recent evidence suggests that they can also infect fungi, such as *Agaricus bisporus* [80], further reinforced by the virus we discovered in *Gaeumannomyces tritici*. Moreover, members of the family *Botourmiaviridae* commonly exhibit a broad host range, with viruses closely related to CrBV capable of infecting members of *Eukaryota*, *Viridiplantae*, and *Metazoa*, in addition to fungi (Supplementary Fig. 1). The LsDV identified in this study shared the closest phylogenetic relationship with a virus identified from *Macrotermes carbonarius* in southern Vietnam (17_N1 + N237) [81]. *M. carbonarius* is an open-air foraging species that collects plant litter and wood debris to cultivate fungi in fungal gardens [82], termites may act as vectors, transmitting deltaflexivirus to other fungi. Furthermore, the viruses we identified, typically associated with fungi, also deepen their connections with species from other kingdoms on the tanglegram tree. For example, while *Partitiviridae* are naturally associated with fungi and plants, NcPV also shows close connections with *Metazoa*. In fact, based largely on phylogenetic predictions, various eukaryotic viruses have been found to undergo horizontal transfer between organisms of plants, fungi, and animals [83]. The rice dwarf virus was demonstrated to infect both plant and insect vectors [84]; moreover, plant-infecting rhabdoviruses, tospoviruses, and tenuiviruses are now known to replicate and spread in vector insects and shuttle between plants and animals [85]. Furthermore, Bian et al. demonstrated that plant virus infection in plants enables Cryphonectria hypovirus 1 to undergo horizontal transfer from fungi to plants and other heterologous fungal species [86].

Recent studies have greatly expanded the diversity of mycoviruses [87, 88]. Gilbert et al. [20] investigated publicly available fungal transcriptomes from the subphylum Pezizomycotina, resulting in the detection of 52 novel mycoviruses; Myers et al. [18] employed both culture-based and transcriptome-mining approaches to identify 85 unique RNA viruses across 333 fungi; Ruiz-Padilla et al. identified 62 new mycoviral species from 248 *Botrytis cinerea* field isolates; Zhou et al. identified 20 novel viruses from 90 fungal strains (across four different macrofungi species) [89]. However, compared to these studies, our work identified fewer novel viruses, possibly due to the following reasons: 1) The libraries from the same Bioproject are usually from the same strains (or isolates). Therefore, there is a certain degree of redundancy in the datasets collected for this study. 2) Contigs shorter than 1,500 nt were discarded, potentially resulting in the oversight of short viral molecules. 3) Establishing a threshold of 70% aa sequence identity may also lead to the exclusion of certain viruses. 4) Some poly(A)-enriched RNA-seq libraries are likely to miss non-polyadenylated RNA viral genomes.

Taxonomy is a dynamic science, evolving with improvements in analytical methods and the emergence of new data. Identifying and rectifying incorrect classifications when new information becomes available is an ongoing and inevitable process in today's rapidly expanding field of virology. For instance, in 1975, members of the genera *Rubivirus* and *Alphavirus* were initially grouped under the family *Togaviridae*; however, in 2019, *Rubivirus* was reclassified into the family *Matonaviridae* due to recognized differences in transmission modes and virion structures [90]. Additionally, the conflicts between certain members of the genera *Magoulivirus* and *Gammapartitivirus* mentioned here and their current demarcation criteria (e.g., amino acid identity, nucleotide length thresholds) need to be reconsidered.

Taken together, these findings reveal the potential diversity and novelty within fungal-associated viral communities and discuss the genetic similarities among different fungal-associated viruses. These findings advance our understanding of fungal-associated viruses and suggest the importance of subsequent in-depth investigations into the interactions between fungi and viruses, which will shed light on the important roles of these viruses in the global fungal kingdom.

### Supplementary Information

Supplementary Material 1.

Supplementary Material 2.

Supplementary Material 3.

## Data Availability

The data reported in this paper have been deposited in the GenBase in National Genomics Data Center, Beijing Institute of Genomics, Chinese Academy of Sciences/China National Center for Bioinformation, under accession numbers C_AA066339.1-C_AA066350.1 that are publicly accessible at https://ngdc.cncb.ac.cn/genbase. Please refer to Table 1 for details.
