# Supplementary material for: Discovery of novel RNA viruses through analysis of fungi-associated next-generation sequencing data
Source: BMC Genomics. 2024 May 27;25:517. doi: 10.1186/s12864-024-10432-w (PMC11129472; doi:10.1186/s12864-024-10432-w)
Supplement: Supplementary file 3 — Supplementary Material 3. [file 12864_2024_10432_MOESM3_ESM.pdf]

Supplementary Figure 1. Tanglegram of phylogenetic trees for virus orders/families and their hosts. Lines and branches are color-coded to indicate host clades. The cophylo function in phytools was employed to enhance congruence between the host (left) and virus (right) phylogenies.

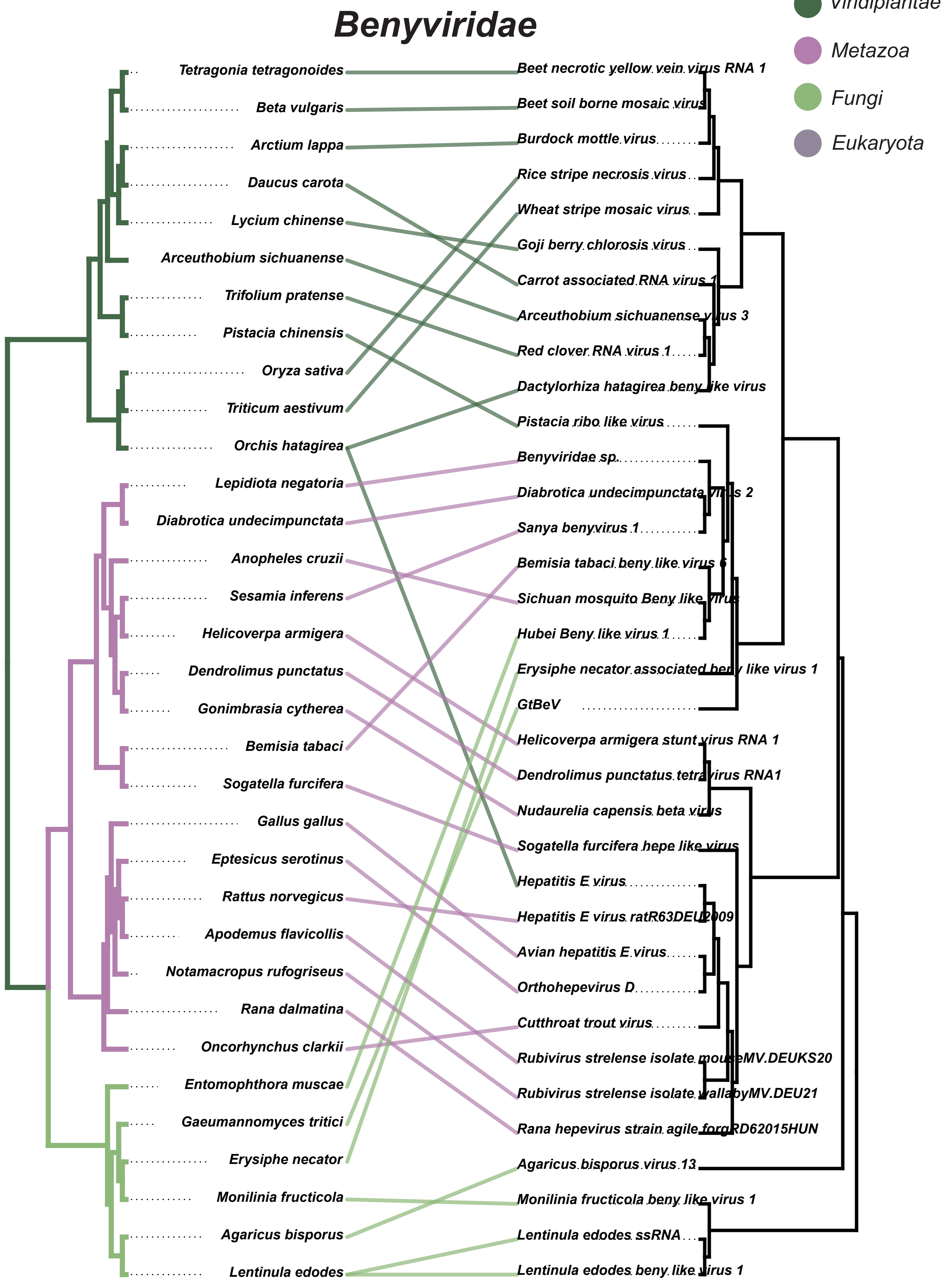

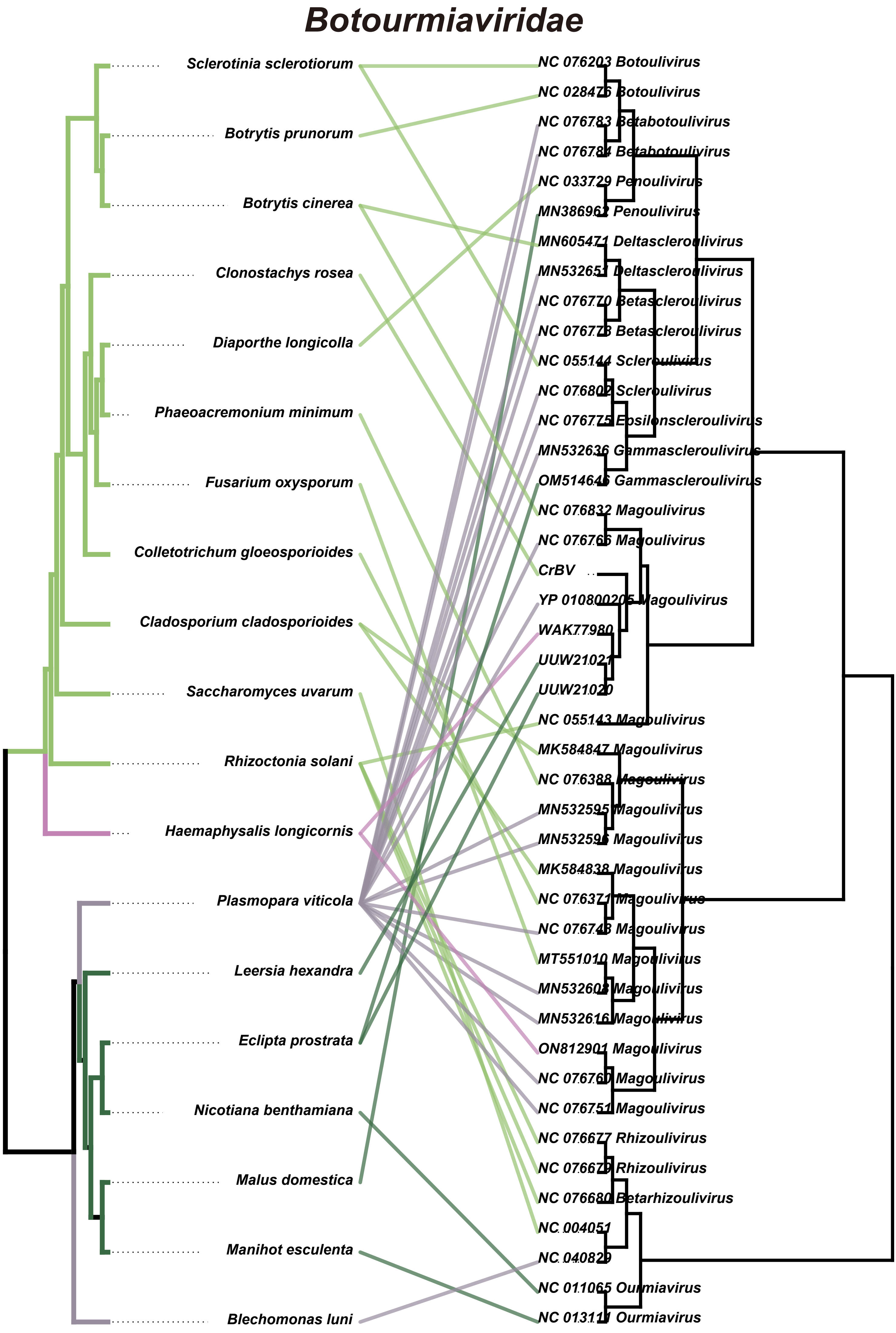
Botourmiaviridae
Sclerotinia sclerotiorum
Botrytis prunorum
Botrytis cinerea
Clonostachys rosea
Diaporthe longicolla
Phaeoacremonium minimum
Fusarium oxysporum
Colletotrichum gloeosporioides
Cladosporium cladosporioides
Saccharomyces uvarum
Rhizoctonia solani
Haemaphysalis longicornis
Plasmopara viticola
Leersia hexandra
Eclipta prostrata
Nicotiana benthamiana
Malus domestica
Manihot esculenta
Blechomonas luni
NC 076203 Botoulivirus
NC 028476 Botoulivirus
NC 076783 Betabotoulivirus
NC 076784 Betabotoulivirus
NC 033729 Penoulivirus
MN386962 Penoulivirus
MN605471 Deltascleroulivirus
MN532651 Deltascleroulivirus
NC 076770 Betascleroulivirus
NC 076778 Betascleroulivirus
NC 055144 Scleroulivirus
NC 076802 Scleroulivirus
NC 076775 Epsilonscleroulivirus
MN532636 Gammascleroulivirus
OM514646 Gammascleroulivirus
NC 076832 Magoulivirus
NC 076766 Magoulivirus
CrBV
YP 010800205 Magoulivirus
WAK77980
UUW21021
UUW21020
NC 055143 Magoulivirus
MK584847 Magoulivirus
NC 076388 Magoulivirus
MN532595 Magoulivirus
MN532596 Magoulivirus
MK584838 Magoulivirus
NC 076371 Magoulivirus
NC 076743 Magoulivirus
MT551010 Magoulivirus
MN532603 Magoulivirus
MN532616 Magoulivirus
ON812901 Magoulivirus
NC 076760 Magoulivirus
NC 076751 Magoulivirus
NC 076677 Rhizoulivirus
NC 076679 Rhizoulivirus
NC 076680 Betarhizoulivirus
NC 004051
NC 040829
NC 011065 Ourmiavirus
NC 013111 Ourmiavirus

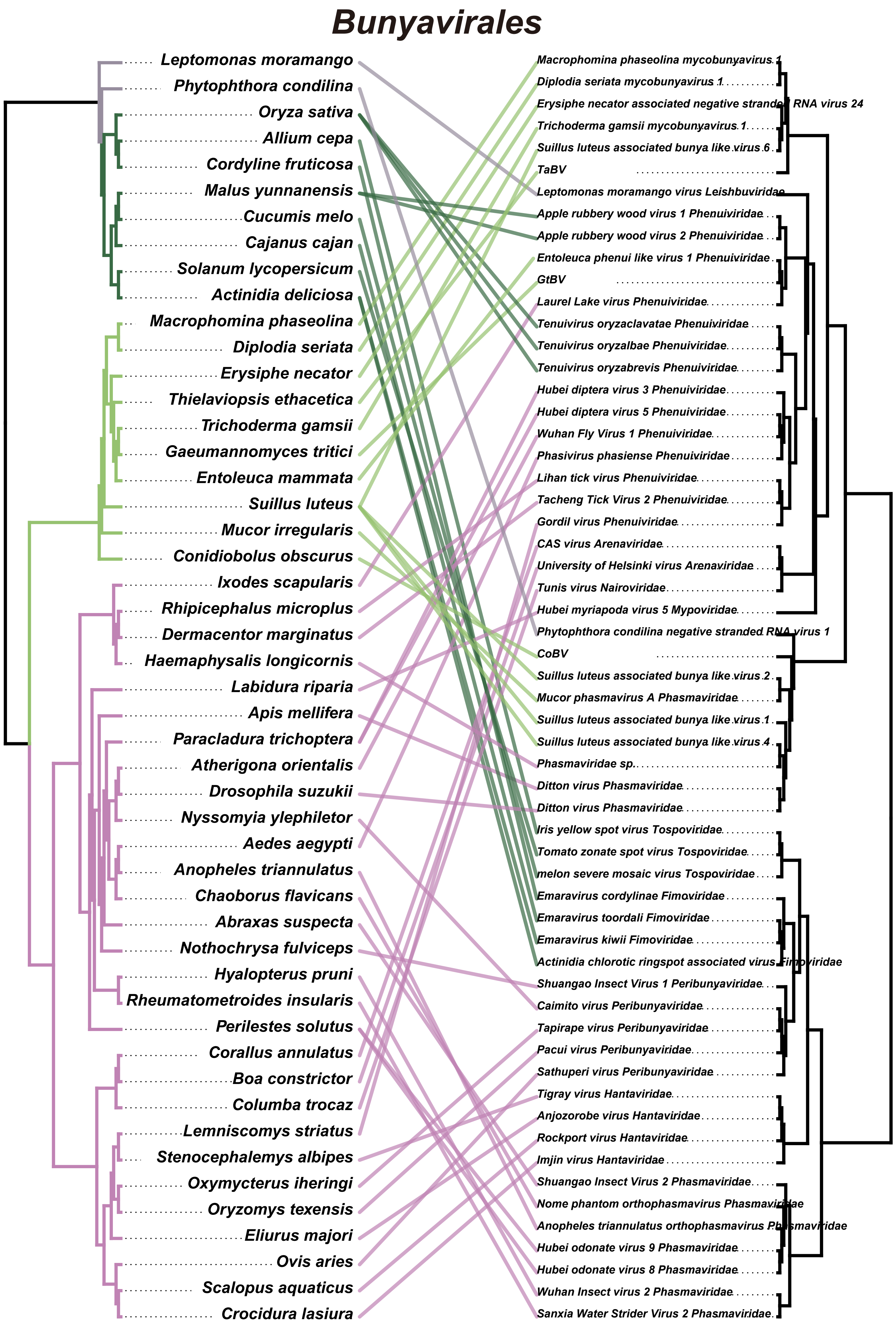
Bunyavirales
Leptomonas moramango
Phytophthora condilina
Oryza sativa
Allium cepa
Cordyline fruticosa
Malus yunnanensis
Cucumis melo
Cajanus cajan
Solanum lycopersicum
Actinidia deliciosa
Macrophomina phaseolina
Diplodia seriata
Erysiphe necator
Thielaviopsis ethacetica
Trichoderma gamsii
Gaeumannomyces tritici
Entoleuca mammata
Suillus luteus
Mucor irregularis
Conidiobolus obscurus
Ixodes scapularis
Rhipicephalus microplus
Dermacentor marginatus
Haemaphysalis longicornis
Labidura riparia
Apis mellifera
Paracladura trichoptera
Atherigona orientalis
Drosophila suzukii
Nyssomyia ylephiletor
Aedes aegypti
Anopheles triannulatus
Chaoborus flavicans
Abraxas suspecta
Nothochrysa fulviceps
Hyalopterus pruni
Rheumatometroides insularis
Perilestes solutus
Corallus annulatus
Boa constrictor
Columba trocaz
Lemniscomys striatus
Stenocephalemys albipes
Oxymycterus iheringi
Oryzomys texensis
Eliurus majori
Ovis aries
Scalopus aquaticus
Crocidura lasiura
Macrophomina phaseolina mycobunyavirus 1
Diplodia seriata mycobunyavirus 1
Erysiphe necator associated negative stranded RNA virus 24
Trichoderma gamsii mycobunyavirus 1
Suillus luteus associated bunya like virus 6
TaBV
Leptomonas moramango virus Leishbuviridae
Apple rubbery wood virus 1 Phenuiviridae
Apple rubbery wood virus 2 Phenuiviridae
Entoleuca phenui like virus 1 Phenuiviridae
GtBV
Laurel Lake virus Phenuiviridae
Tenuivirus oryzaclavatae Phenuiviridae
Tenuivirus oryzalbae Phenuiviridae
Tenuivirus oryzabrevis Phenuiviridae
Hubei diptera virus 3 Phenuiviridae
Hubei diptera virus 5 Phenuiviridae
Wuhan Fly Virus 1 Phenuiviridae
Phasivirus phasiense Phenuiviridae
Lihan tick virus Phenuiviridae
Tacheng Tick Virus 2 Phenuiviridae
Gordil virus Phenuiviridae
CAS virus Arenaviridae
University of Helsinki virus Arenaviridae
Tunis virus Nairoviridae
Hubei myriapoda virus 5 Mypoviridae
Phytophthora condilina negative stranded RNA virus 1
CoBV
Suillus luteus associated bunya like virus 2
Mucor phasmavirus A Phasmaviridae
Suillus luteus associated bunya like virus 1
Suillus luteus associated bunya like virus 4
Phasmaviridae sp.
Ditton virus Phasmaviridae
Ditton virus Phasmaviridae
Iris yellow spot virus Tospoviridae
Tomato zonate spot virus Tospoviridae
melon severe mosaic virus Tospoviridae
Emaravirus cordylinae Fimoviridae
Emaravirus toordali Fimoviridae
Emaravirus kiwii Fimoviridae
Actinidia chlorotic ringspot associated virus Fimoviridae
Shuangao Insect Virus 1 Peribunyaviridae
Caimito virus Peribunyaviridae
Tapirape virus Peribunyaviridae
Pacui virus Peribunyaviridae
Sathuperi virus Peribunyaviridae
Tigray virus Hantaviridae
Anjozorobe virus Hantaviridae
Rockport virus Hantaviridae
Imjin virus Hantaviridae
Shuangao Insect Virus 2 Phasmaviridae
Nome phantom orthophasmavirus Phasmaviridae
Anopheles triannulatus orthophasmavirus Phasmaviridae
Hubei odonate virus 9 Phasmaviridae
Hubei odonate virus 8 Phasmaviridae
Wuhan Insect virus 2 Phasmaviridae
Sanxia Water Strider Virus 2 Phasmaviridae

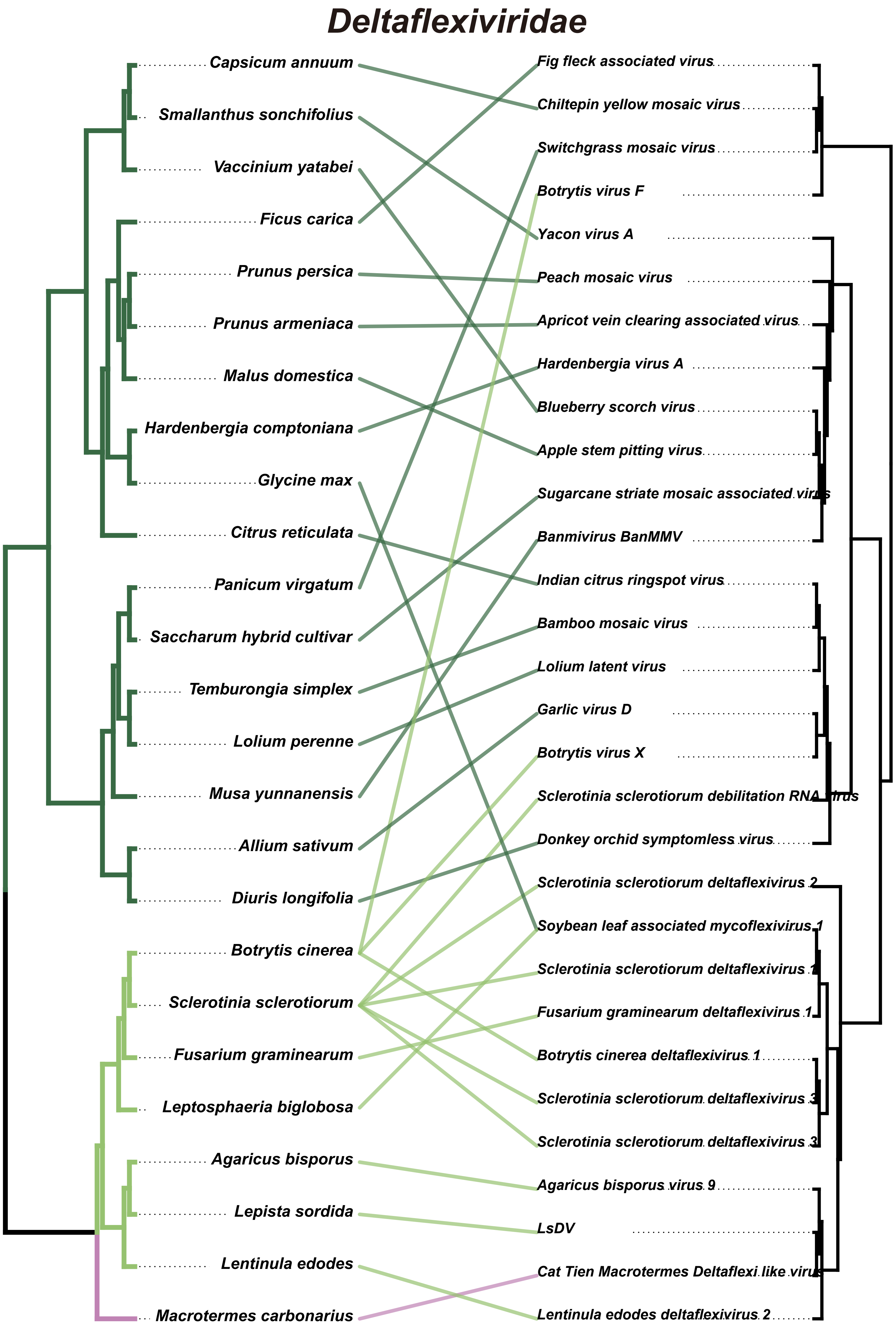

Deltaflexiviridae
Capsicum annuum
Smallanthus sonchifolius
Vaccinium yatabei
Ficus carica
Prunus persica
Prunus armeniaca
Malus domestica
Hardenbergia comptoniana
Glycine max
Citrus reticulata
Panicum virgatum
Saccharum hybrid cultivar
Temburongia simplex
Lolium perenne
Musa yunnanensis
Allium sativum
Diuris longifolia
Botrytis cinerea
Sclerotinia sclerotiorum
Fusarium graminearum
Leptosphaeria biglobosa
Agaricus bisporus
Lepista sordida
Lentinula edodes
Macrotermes carbonarius
Fig fleck associated virus
Chiltepin yellow mosaic virus
Switchgrass mosaic virus
Botrytis virus F
Yacon virus A
Peach mosaic virus
Apricot vein clearing associated virus
Hardenbergia virus A
Blueberry scorch virus
Apple stem pitting virus
Sugarcane striate mosaic associated virus
Banmivirus BanMMV
Indian citrus ringspot virus
Bamboo mosaic virus
Lolium latent virus
Garlic virus D
Botrytis virus X
Sclerotinia sclerotiorum debilitation RNA virus
Donkey orchid symptomless virus
Sclerotinia sclerotiorum deltaflexivirus 2
Soybean leaf associated mycoflexivirus 1
Sclerotinia sclerotiorum deltaflexivirus 1
Fusarium graminearum deltaflexivirus 1
Botrytis cinerea deltaflexivirus 1
Sclerotinia sclerotiorum deltaflexivirus 3
Sclerotinia sclerotiorum deltaflexivirus 3
Agaricus bisporus virus 9
LsDV
Cat Tien Macrotermes Deltaflexi like virus
Lentinula edodes deltaflexivirus 2

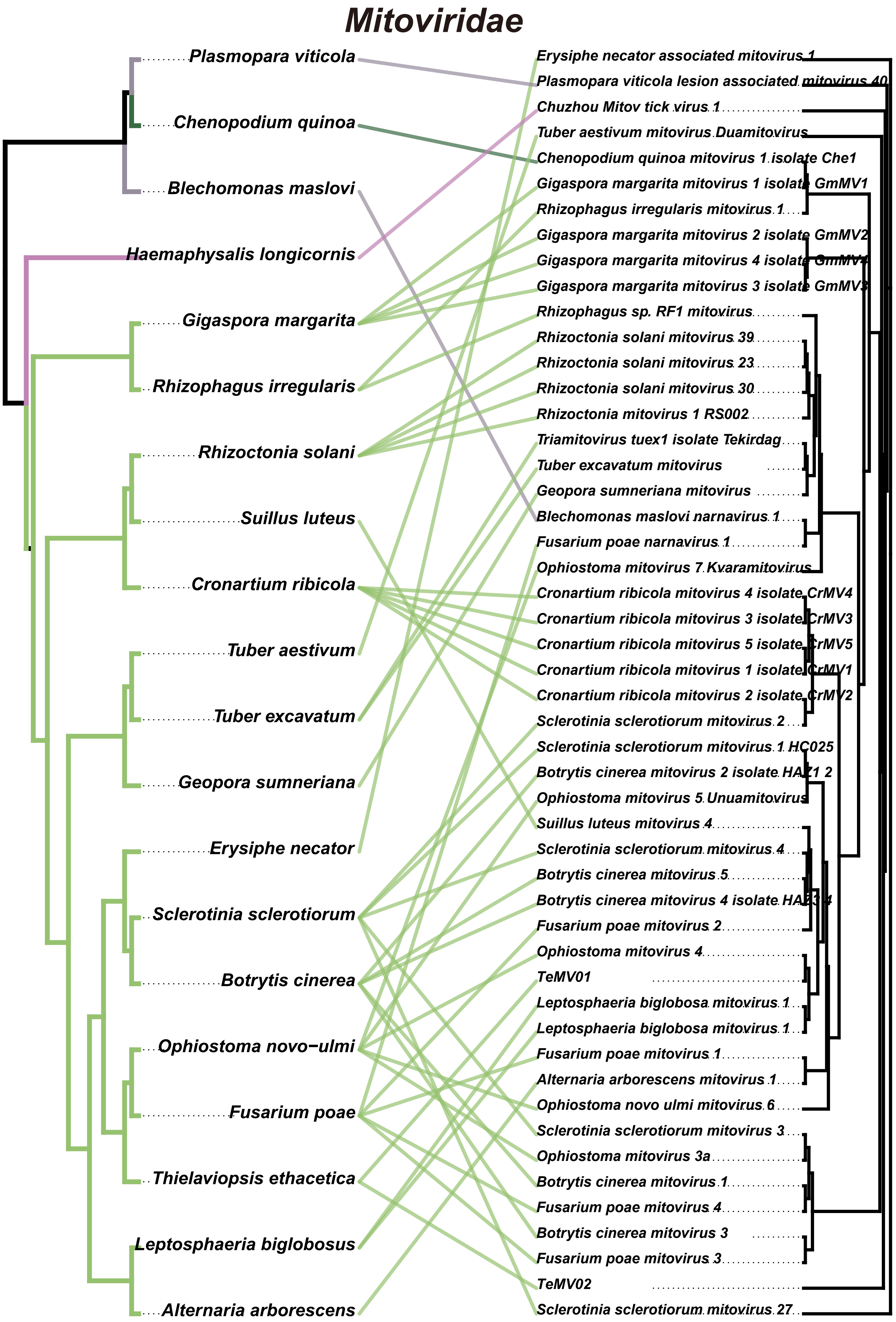
Mitoviridae
Plasmopara viticola
Chenopodium quinoa
Blechomonas maslovi
Haemaphysalis longicornis
Gigaspora margarita
Rhizophagus irregularis
Rhizoctonia solani
Suillus luteus
Cronartium ribicola
Tuber aestivum
Tuber excavatum
Geopora sumneriana
Erysiphe necator
Sclerotinia sclerotiorum
Botrytis cinerea
Ophiostoma novo−ulmi
Fusarium poae
Thielaviopsis ethacetica
Leptosphaeria biglobosus
Alternaria arborescens
Erysiphe necator associated mitovirus 1
Plasmopara viticola lesion associated mitovirus 40
Chuzhou Mitov tick virus 1
Tuber aestivum mitovirus Duamitovirus
Chenopodium quinoa mitovirus 1 isolate Che1
Gigaspora margarita mitovirus 1 isolate GmMV1
Rhizophagus irregularis mitovirus 1
Gigaspora margarita mitovirus 2 isolate GmMV2
Gigaspora margarita mitovirus 4 isolate GmMV4
Gigaspora margarita mitovirus 3 isolate GmMV3
Rhizophagus sp. RF1 mitovirus
Rhizoctonia solani mitovirus 39
Rhizoctonia solani mitovirus 23
Rhizoctonia solani mitovirus 30
Rhizoctonia mitovirus 1 RS002
Triamitovirus tuex1 isolate Tekirdag
Tuber excavatum mitovirus
Geopora sumneriana mitovirus
Blechomonas maslovi narnavirus 1
Fusarium poae narnavirus 1
Ophiostoma mitovirus 7 Kvaramitovirus
Cronartium ribicola mitovirus 4 isolate CrMV4
Cronartium ribicola mitovirus 3 isolate CrMV3
Cronartium ribicola mitovirus 5 isolate CrMV5
Cronartium ribicola mitovirus 1 isolate CrMV1
Cronartium ribicola mitovirus 2 isolate CrMV2
Sclerotinia sclerotiorum mitovirus 2
Sclerotinia sclerotiorum mitovirus 1 HC025
Botrytis cinerea mitovirus 2 isolate HAZ1 2
Ophiostoma mitovirus 5 Unuamitovirus
Suillus luteus mitovirus 4
Sclerotinia sclerotiorum mitovirus 4
Botrytis cinerea mitovirus 5
Botrytis cinerea mitovirus 4 isolate HAZ3 4
Fusarium poae mitovirus 2
Ophiostoma mitovirus 4
TeMV01
Leptosphaeria biglobosa mitovirus 1
Leptosphaeria biglobosa mitovirus 1
Fusarium poae mitovirus 1
Alternaria arborescens mitovirus 1
Ophiostoma novo ulmi mitovirus 6
Sclerotinia sclerotiorum mitovirus 3
Ophiostoma mitovirus 3a
Botrytis cinerea mitovirus 1
Fusarium poae mitovirus 4
Botrytis cinerea mitovirus 3
Fusarium poae mitovirus 3
TeMV02
Sclerotinia sclerotiorum mitovirus 27

Mymonaviridae

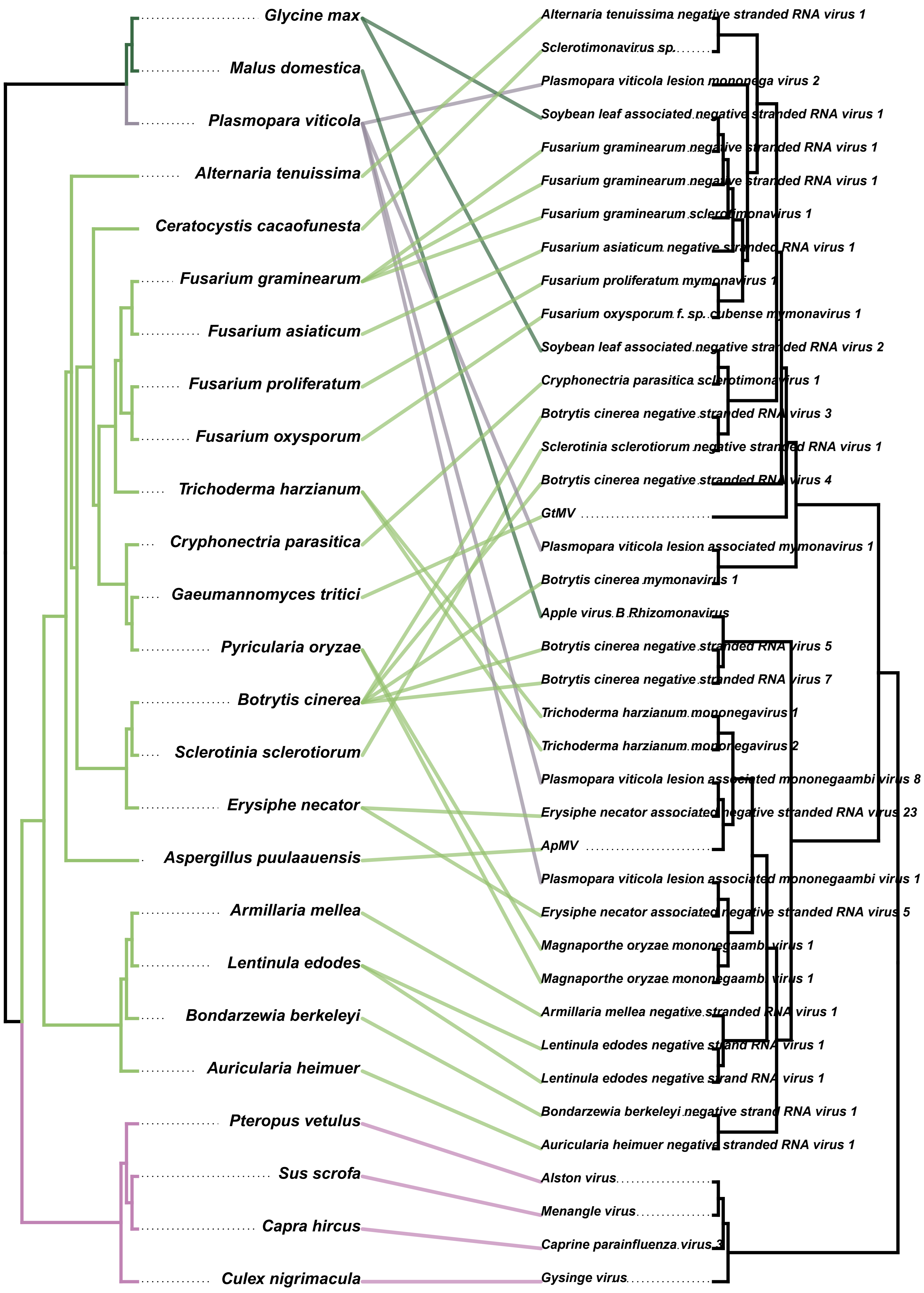

Partitiviridae

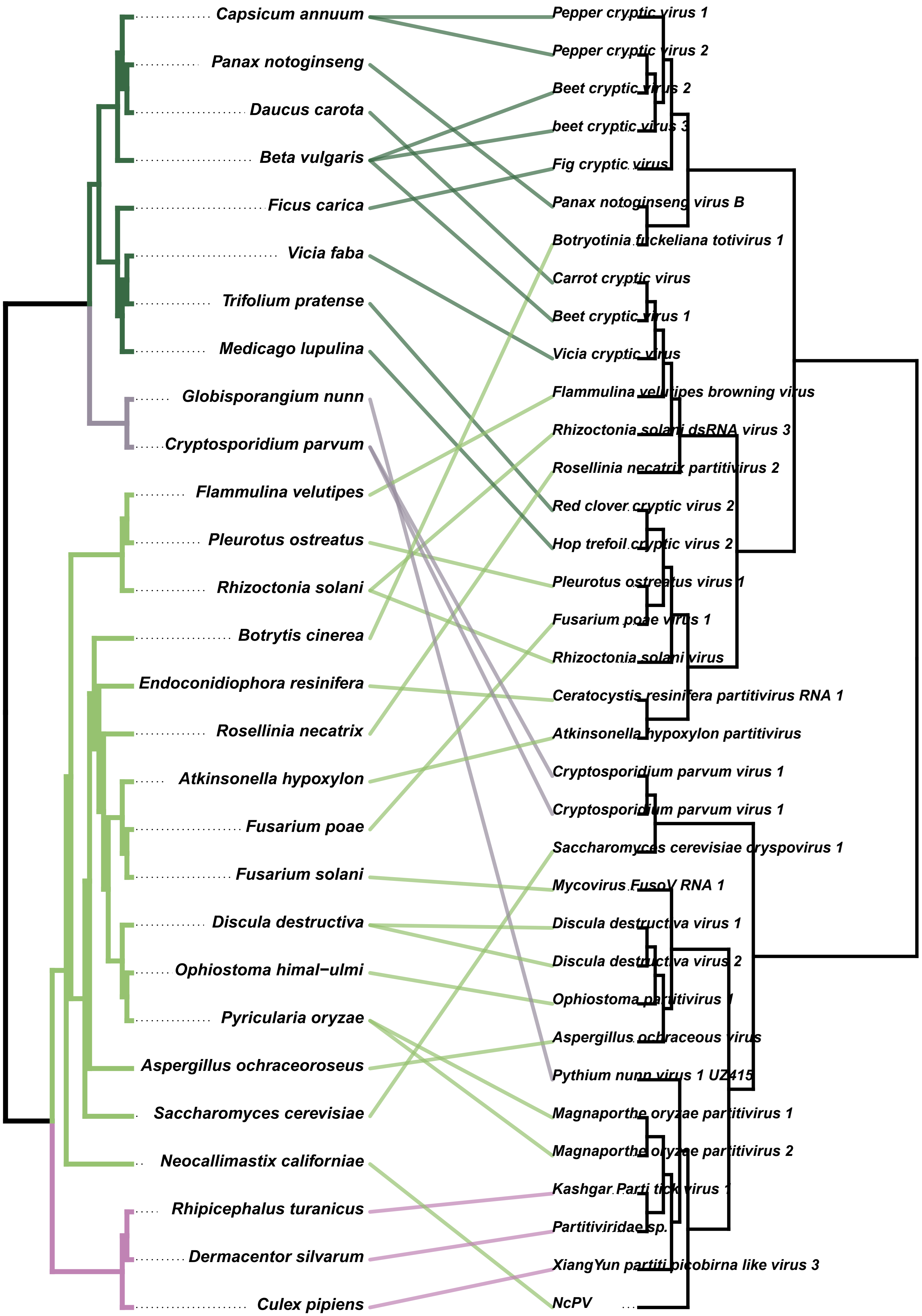

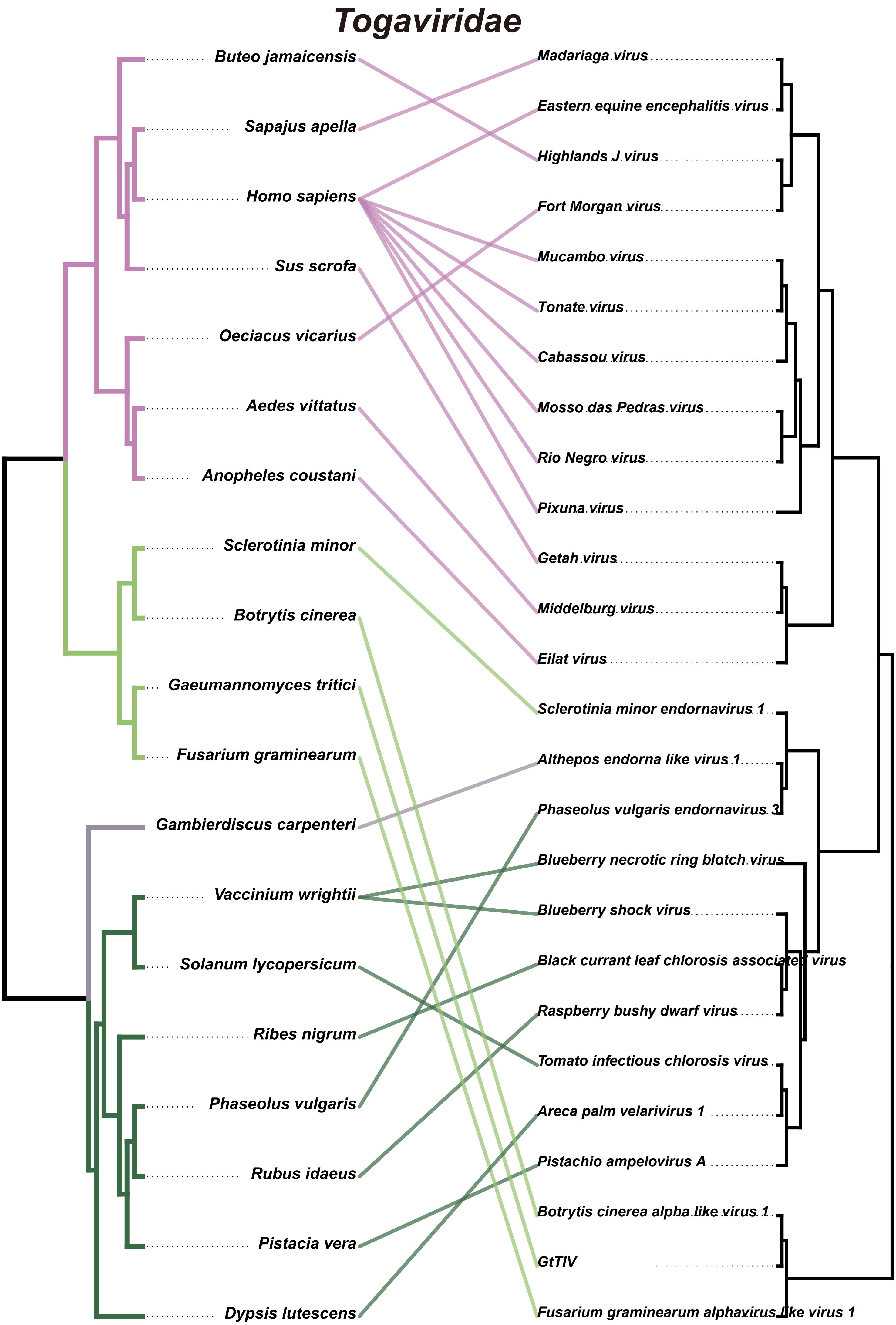

Togaviridae
Buteo jamaicensis
Sapajus apella
Homo sapiens
Sus scrofa
Oeciacus vicarius
Aedes vittatus
Anopheles coustani
Sclerotinia minor
Botrytis cinerea
Gaeumannomyces tritici
Fusarium graminearum
Gambierdiscus carpenteri
Vaccinium wrightii
Solanum lycopersicum
Ribes nigrum
Phaseolus vulgaris
Rubus idaeus
Pistacia vera
Dypsis lutescens
Madariaga virus
Eastern equine encephalitis virus
Highlands J virus
Fort Morgan virus
Mucambo virus
Tonate virus
Cabassou virus
Mosso das Pedras virus
Rio Negro virus
Pixuna virus
Getah virus
Middelburg virus
Eilat virus
Sclerotinia minor endornavirus 1
Althepos endorna like virus 1
Phaseolus vulgaris endornavirus 3
Blueberry necrotic ring blotch virus
Blueberry shock virus
Black currant leaf chlorosis associated virus
Raspberry bushy dwarf virus
Tomato infectious chlorosis virus
Areca palm velarivirus 1
Pistachio ampelovirus A
Botrytis cinerea alpha like virus 1
GtTIV
Fusarium graminearum alphavirus like virus 1
